# Characteristics of sound localization in children with unilateral microtia and atresia and predictors of localization improvement when using a bone conduction device

**DOI:** 10.3389/fnins.2022.973735

**Published:** 2022-08-25

**Authors:** Yujie Liu, Chunli Zhao, Lin Yang, Peiwei Chen, Jinsong Yang, Danni Wang, Ran Ren, Ying Li, Shouqin Zhao, Shusheng Gong

**Affiliations:** ^1^Ministry of Education Key Laboratory of Otolaryngology Head and Neck Surgery, Department of Otolaryngology Head and Neck Surgery, Beijing Tongren Hospital, Capital Medical University, Beijing, China; ^2^Department of Otolaryngology Head and Neck Surgery, Beijing Friendship Hospital, Capital Medical University, Beijing, China

**Keywords:** unilateral, microtia and atresia, congenital conductive hearing loss, speech perception, sound localization, bone conduction device

## Abstract

This study aimed to determine the characteristics of sound localization in children with unilateral microtia and atresia (UMA) and the influence of a non-surgical bone conduction device (BCD). Hearing benefits were evaluated by the word recognition score (WRS), speech reception threshold, the international outcome inventory for hearing aids (IOI-HA), and the Speech, Spatial, and Qualities of Hearing Test for Parent (SSQ-P). Sound localization was measured using broadband noise stimuli randomly played from seven loudspeakers at different stimulus levels [65, 70, and 75 dB sound pressure levels (SPLs)]. The average unaided WRS and speech-to-noise ratio (SNR) for UMA patients was 18.27 ± 14.63 % and −5 ± 1.18 dB SPL, and the average aided WRS and SNR conspicuously changed to 85.45 ± 7.38 % and −7.73 ± 1.42 dB SPL, respectively. The mean IOI-HA score was 4.57 ± 0.73. Compared to the unaided condition, the mean SSQ-P score in each domain improved from 7.08 ± 2.5, 4.86 ± 2.27, and 6.59 ± 1.4 to 8.72 ± 0.95, 7.61 ± 1.52, and 8.55 ± 1.09, respectively. In the sound localization test, some children with UMA were able to detect sound sources quite well and the sound localization abilities did not deteriorate with the non-surgical BCD. Our study concludes that for children with UMA, the non-surgical BCD provided a definite benefit on speech recognition and high satisfaction without deteriorating their sound localization abilities. It is an efficient and safe solution for the early hearing intervention of these patients.

## Introduction

Hearing loss is a major global problem and was described as an epidemic of the twenty-first century by the World Health Organization [WHO] (2021). Hearing loss can be congenital or acquired, with possible etiologies including genetic causes (Hong et al., 2022; Tao et al., 2022), infections (Shahar-Nissan et al., 2022), excessive noise (Jiang et al., 2021), ototoxic drugs (Fu et al., 2022; Wang et al., 2022; Zhang et al., 2022), and aging (He et al., 2021). For patients with profound bilateral hearing impairment, treatment is always offered early in life, but for patients with unilateral form, the hearing intervention tends to be delayed, as the normal hearing (NH) ear provides enough hearing cues for basic speech understanding. However, functional deficits of disability in speech recognition and inaccuracy of sound localization have been reported in patients with unilateral hearing loss, and they may also experience an apparent handicap in academic performance and social interactions (van Wieringen et al., 2019; Okada et al., 2020).

Microtia and atresia, a developmental malformation of the middle and external ear, is a common cause of congenital conductive hearing loss. Two-thirds of patients with microtia and atresia experience the unilateral form [i.e., unilateral microtia and atresia (UMA)] with unilateral conductive hearing loss (UCHL) (Luquetti et al., 2012; Bartel-Friedrich, 2015). Common treatment options for patients with UMA include traditional canaloplasty, active middle ear implants, bone conduction implants, and non-surgical bone conduction devices (BCDs). For children with UMA who are not willing to undergo surgery or who have not reached the age for surgery, non-surgical BCDs represent an important transition intervention (Liu et al., 2017). Non-surgical BCDs can provide evident speech recognition-related benefits to patients with UCHL; however, whether these patients can achieve more accurate sound localization after receiving such interventions remains disputed (Kunst et al., 2008; Yu et al., 2014; Liu et al., 2017; Vyskocil et al., 2017). Thus, doctors and parents often face the dilemma of whether a BCD should be selected for these children at an early age.

Sound localization is a complex process that relies on the computation and integration of multiple spatial cues at the level of the auditory pathway (Tillein et al., 2016; Risoud et al., 2018; Wood et al., 2019). For patients having acquired UCHL with a mature auditory system, definite improvement of sound localization ability was observed after hearing intervention (Agterberg et al., 2011, 2012). Regarding congenital UCHL, the results seem to be contentious. Some studies have reported remarkable improvements in horizontal spatial hearing in patients with congenital UCHL aided with BCD, despite the inherent problems of time delay and cross-hearing (Nelissen et al., 2016; Vyskocil et al., 2017). In contrast, other studies have suggested that congenital UCHL cannot benefit from BCDs in terms of horizontal spatial hearing abilities (Kunst et al., 2008; Weiss et al., 2017). They maintained that listeners with congenital UCHL might have adapted to their hearing impairment as they learned to rely on the spectral shape cues and ambiguous monaural head shadow effect (HSE) cues, which had developed during the long-term unilateral hearing deprivation (Van Wanrooij and Van Opstal, 2004; Vogt et al., 2020). When aided with a BCD, such listening cues might be distorted sharply, thus jeopardizing the original directional hearing. Given the uncertain benefits of hearing amplification and non-aesthetic reasons, studies concerning the sound localization ability of children with UMA are limited by heterogeneous patient populations, varying in study design and audiological test results. How bone conduction (BC) stimulation affects spatial hearing abilities and the predictive factors that may affect the degree of the benefit provided by BCDs are still unknown.

Currently, there is no research investigating the characteristics of sound localization and the effects of non-surgical BCDs in school-aged children with UMA. This study had three primary objectives: to detect the hearing benefits of a BCD on speech perception and subjective satisfaction in children with congenital UMA; to compare characteristics of sound localization in children with congenital UMA and children with NH, as well as acquired UCHL; to investigate whether the use of BCD would be detrimental to the original sound localization of children with UMA and reveal predictive factors for the improvement of sound localization accuracy after using a BCD.

## Materials and methods

### Ethics

Ethical approval was given by the medical committee of Beijing Tongren Hospital, Capital Medical University (TRECKY2018-067). Written informed consent for participation was obtained from the parents and guardians of the participants.

### Participants

Eleven children (mean ± SD: 7.45 ± 1.81 years) who had UMA and congenital UCHL were included. All patients had NH in one ear [hearing thresholds, ≤ 25 dB hearing level (HL) across 0.5–4 kHz] and pure conductive hearing loss in the impaired ear (air-bone gap, ≥ 25 dB HL, BC thresholds of ≤ 25 dB HL across 0.25–4 kHz). For comparative purposes, eight boys and three girls aged 6–12 years who had bilateral NH were recruited as control listeners. All control listeners had bilateral air and BC hearing thresholds ≤ 25 dB HL across frequencies of 0.5–4 kHz. Detailed demographic data are summarized in Table 1.

**TABLE 1:** The demographic data of 11 patients with UMA and 11 children with NH.

Participant number	Sex	Age (years)	Side of impaired/plugged	Etiology	Follow-up time (weeks)
P1	M	9	R	Atresia	8
P2	F	7	R	Atresia	13
P3	M	8	L	Stenosis	9
P4	M	8	R	Atresia	8
P5	F	5	L	Atresia	8
P6	M	7	R	Atresia	12
P7	F	11	L	Stenosis	8
P8	M	6	R	Atresia	9
P9	M	7	L	Atresia	8
P10	M	5	R	Stenosis	11
P11	M	9	L	Atresia	8
Mean ± *SD*	–	7.45 ± 1.81			9.27 ± 1.85
N1	M	8	R	–	–
N2	M	9	L	–	–
N3	F	11	R	–	–
N4	M	11	R	–	–
N5	F	7	L	–	–
N6	M	9	R	–	–
N7	F	6	L	–	–
N8	M	7	R	–	–
N9	M	8	L	–	–
N10	M	12	L	–	–
N11	M	10	R	–	–
Mean ± *SD*	–	8.91 ± 1.92	–	–	–

### Device and listening conditions

The BCD used in the current study was a non-surgical adhesive device (ADHEAR; MED-EL, Innsbruck, Austria). All devices were set in the omnidirectional mode for all experimental conditions, and the volume was adjusted based on patients’ preferences. The system fittings of the ADHEAR did not change during all experiments.

**FIGURE 1:**
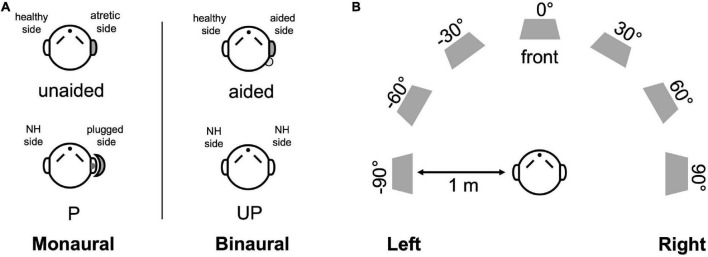
Test setup and listening conditions. **(A)** The monaural (left) and binaural listening (right) conditions are designed for UMA patients (unaided and aided conditions) and controls (P and UP conditions). **(B)** Seven loudspeakers were placed at 30^°^ intervals in a semicircle in a double-walled, soundproof laboratory. NH, normal hearing; P, the plugged condition; UP, the unplugged condition.

Children with UMA were tested with the BCD off (unaided condition) and on (aided condition) (Figure 1A). Children with NH were measured with both ears unplugged (UP condition) as normal controls. When measuring sound localization, control listeners were also tested with plugging (P condition) to stimulate an acquired UCHL to reveal the difference in directional hearing between the acquired UCHL and UMA (congenital UCHL). Plugging was performed by covering an ear with an earmuff (Peltor X5A; 3M Company, MN, United States), along with a foam earplug (E-A-R soft; 3M Company, MN, United States) inserted into the external auditory canal. The plugging provided a mean attenuation of 40.22 dB ± 2.29 dB HL, from 0.5 to 4 kHz (measured by audiometric threshold shifts) in the sound field.

For the UMA group, unaided audiological tests were performed on the day they received the BCD and aided audiological tests were measured after a mean period of 9.27 ± 1.85 weeks. For the NH group, all tests were performed in one visit.

### Setup and stimuli

All tests were conducted in a double-walled soundproof laboratory. Participants sat in a chair placed 1 m in front of seven loudspeakers. Sound field hearing thresholds were obtained by warble tones for octave frequencies across 0.25–4 kHz in dB HL. Speech perception under quiet was measured by the word recognition score [WRS (%)] of the Mandarin speech test materials (MSTMs) (Wang et al., 2007) at 65 dB sound pressure level (SPL). Speech perception in noise was measured by the speech reception threshold (SRT) of the MSTMs. The spectrum-shaped noise (SSN) was set at 65 dB SPL, and the speech signal started at 0 dB speech-to-noise ratio (SNR), with the following disyllables changing adaptively in 2 dB SPL steps as the participants responded. The SRT was defined as the speech signal level presented when a participant identified 50% of the words correctly. The SNR was calculated as the difference between the SRT and SSN.

Sound localization was measured in a double-walled soundproof laboratory with seven audiometric loudspeakers placed at 30° intervals in a semicircle within the horizontal plane (± 90^°^, azimuth) (Figure 1B). Broadband noise (0.5–20 kHz), with a duration of 1 s, was randomly played at three different sound levels (65, 70, and 75 dB SPL). During the test, each loudspeaker was randomly presented twice at each sound level burst; thus, 42 stimuli were included in each test. The participants sat comfortably in a chair located 1 m in front of the loudspeaker, facing and fixating the loudspeaker at 0^°^, azimuth. They were not permitted to move their heads when the noise bursts were presented. After the loudspeaker finished each presentation, participants were allowed to indicate the orientation and could turn their heads to look at the number of the loudspeakers that they considered to be the source of the burst.

To familiarize the participants with the experiment before the formal sound localization tests, a brief block of 12 broadband stimuli was presented. They were instructed to localize the stimuli as fast as possible, and no feedback was provided throughout the training to avoid the influence of learning in the formal test.

### Subject satisfaction

The subjective satisfaction was measured with two questionnaires, the Chinese version of the International Outcome Inventory for Hearing Aids (IOI-HA) (Liu et al., 2011) and the Speech, Spatial, and Qualities of Hearing Test for Parent (SSQ-P) (Gao et al., 2022), which were handed out to patients’ parents at the end of the follow-up. The IOI-HA consists of seven items: daily use, benefit, residual activity limitations, satisfaction, residual participation restrictions, impact on others, and quality of life. Each answer is rated on a scale from 0 to 5, with higher ratings reflecting better outcomes (or fewer residual difficulties). The SSQ-P across three domains: speech, spatial hearing, and qualities of hearing, with higher scores in each subdomain representing higher satisfaction. To evaluate the hearing impairment of patients with UMA in unaided conditions, the SSQ-P was also handed out to their parents before they were equipped with the ADHEAR.

### Data analysis


(1) $$M\,A\,E=\frac{\sum_{i=1}^{n}\left|\alpha \,\begin{array}{c} i\\ R\,E\,S\,P \end{array}-\alpha \,\begin{array}{c} i\\ T\,A\,R\,G \end{array}\right|}{n}$$

(2) $$\alpha_{R\,E\,S\,P}=g\,a\,i\,n\cdot \alpha_{T\,A\,R\,G}+b$$

The mean absolute error (MAE) was calculated using Equation 1 to assess the sound localization accuracy under different conditions, where the α_*RESP*_ and α_*TARG*_ referred to the response azimuth and target azimuth (both in degrees). Additionally, the best linear fit of the target-response relationship for each participant was also computed using Equation 2, where *g* is the response gain (slope, dimensionless), and *b* is the response bias (offset in degrees). In this study, the right side was defined as the impaired side; therefore, azimuth coordinates for patients with left ear impairment and controls with NH with left ears plugged were inverted.

Paired and independent *t*-tests were conducted to evaluate differences under different test conditions. The Mann-Whitney *U*-test was used to compare the difference in the unaided, aided, and delta MAEs (delta MAE = aided MAE - unaided MAE) between the groups of different sexes, sides of impairment, and etiologies. Spearman correlation analysis was conducted to analyze the correlations between continuous variables (age and follow-up time) and the unaided, aided, and delta MAEs, respectively. The *p*-values of < 0.05 and < 0.01 were considered statistically significant. SPSS version 26.0 (IBM Corp., Armonk, NY, United States) and GraphPad Prism version 8.0 (GraphPad, San Diego, CA) were used to analyze the data and draw diagrams.

## Results

### Hearing benefits

**FIGURE 2:**
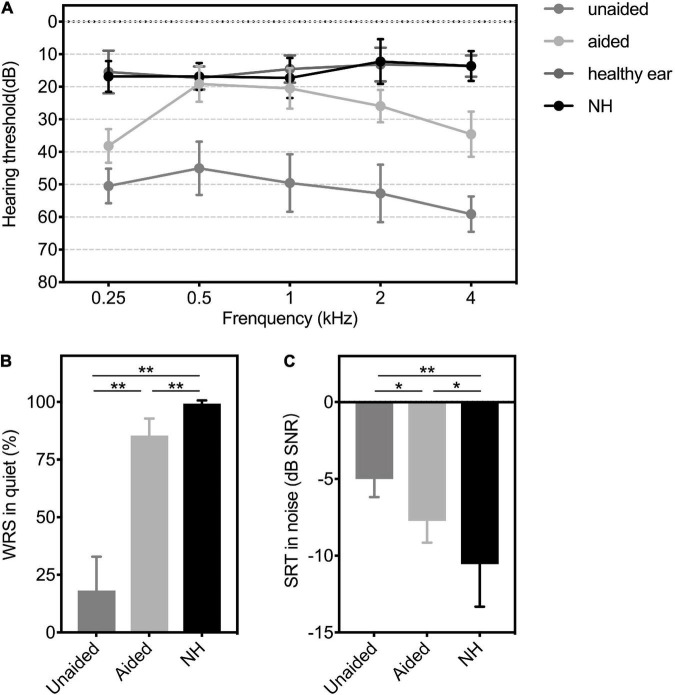
**(A)** Hearing thresholds, **(B)** WRS in quiet, and **(C)** SRT in the noise of patients with UMA in unaided and aided conditions, as well as the controls. Group means are presented as mean ± *SD*. Significant differences, *p < 0.05, **p < 0.01. WRS, word recognition score; SRT, speech reception threshold; SNR, speech-to-noise ratio; *SD*, standard deviation; Unaided, the unaided condition of UMA; Aided, the aided condition of UMA; healthy ear: the healthy ear of patients with UMA; NH, normal hearing group.

For patients with UMA, the mean hearing threshold of the healthy ear was 14.82 ± 3.82 dB HL. The unaided hearing threshold was 51.36 ± 5.02 dB HL and significantly improved to 27.64 ± 2.38 dB HL with a mean functional hearing gain (FHG) of 23.73 ± 3.47 dB HL (*p* < 0.01). For the NH comparison group, the mean hearing threshold was 15.36 ± 3.88 dB HL. The aided mean hearing threshold of the UMA group was still higher (worse) than the mean hearing threshold of the NH group (*p* < 0.01). Detailed outcomes across 0.5–4 kHz are depicted in Figure 2A.

The average unaided WRS and SNR for patients with UMA were 18.27 ± 14.63 % and −5 ± 1.18 dB SPL, respectively, whereas the average aided WRS and SNR conspicuously changed to 85.45 ± 7.38 % and −7.73 ± 1.42 dB SPL, respectively (WRS: *p* < 0.01; SNR: *p* < 0.05). The mean WRS and SNR of the comparison group were 99.27 ± 1.35 % and −10.55 ± 2.77 dB SPL, respectively. Figures 2B,C show significant differences in the speech levels between patients aided with BCDs and their peers with NH (WRS: *p* < 0.01; SNR: *p* < 0.05).

**FIGURE 3:**
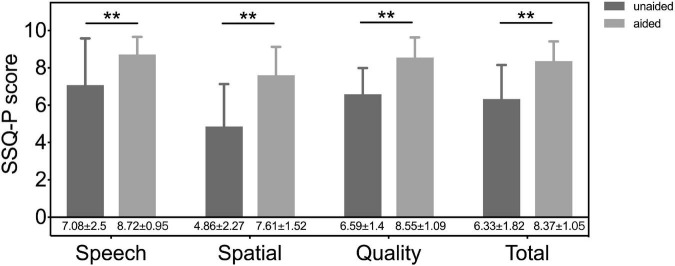
The results of speech, spatial and qualities of hearing scale for parents (SSQ-p) without (unaided) and with (aided) a BCD. Significant differences, **p < 0.01. Unaided, the unaided condition of UMA; aided, the aided condition of UMA.

The mean overall IOI-HA score was 4.57 ± 0.73. A score > 3 per item, defined as a benefit from the BCD, was found for nearly all participants. The mean score for items 1–7 were 3.86 ± 0.31, 3.57 ± 0.25, 4.29 ± 0.19, 3.79 ± 0.24, 4.14 ± 0.14, 4.64 ± 0.13, and 4 ± 0.23, respectively. The results of the SSQ-P without (unaided) and with (aided) BCD are presented in Figure 3, and significant improvements of subjective satisfaction with the ADHEAR were found in each subdomain and the total rating (all *p* < 0.01).

### Sound localization in patients with unilateral microtia and atresia and stimulated unilateral conductive hearing loss

**FIGURE 4:**
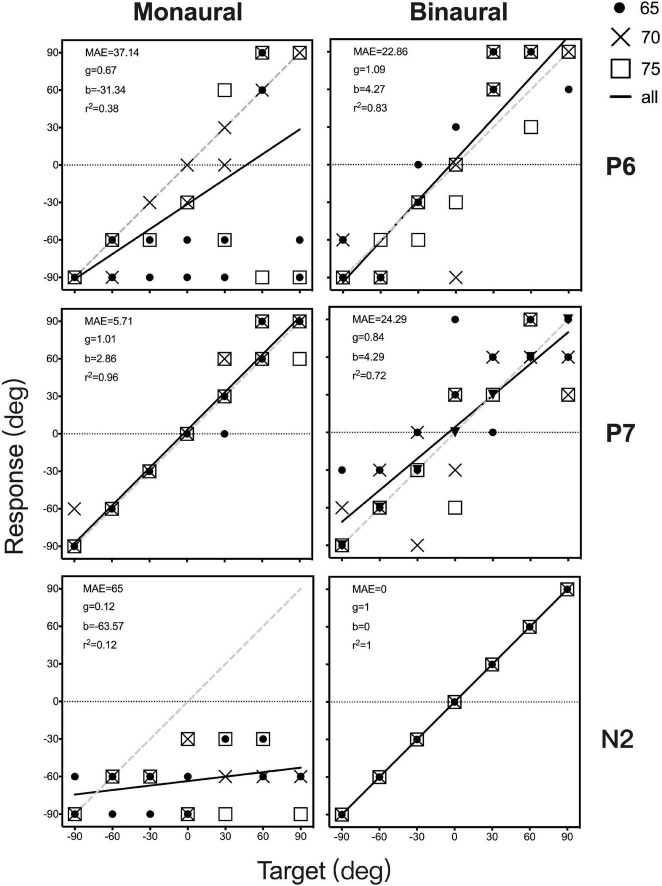
Sound localization target-response plots of two patients (P6 and P7) and one control (N2) in monaural (unaided and P, left) and binaural (aided and UP, right) listening conditions. Stimulus sound levels are indicated by black circle (65 dB SPL), and cross data (70 dB SPL), and white square points (75 dB SPL). Best-fit linear regression is indicated by a black line. For participants with an ideal optimal localization ability, gain is 1, whereas MAE and *b* are 0. MAE, mean absolute error; g, response gain; b, response bias; *r*^2^, R square.

Figure 4 shows the individual sound localization target-response plots for two children with UMA (P6 and P7) and one control (N2) under monaural (unaided and P, left column) and binaural (aided and UP, right column) listening conditions. Under the unaided condition, P6 showed a poor localization ability and perceived most stimuli from the healthy ear side. However, P7 exhibited relatively better sound localization accuracy than P6. When aided with the BCD, the sound localization accuracy improved in P6 (delta gain = 0.422, delta MAE = -14.28°), and the application of the BCD led to a decrease in sound localization accuracy in P7 (delta gain = -0.167, delta MAE = 18.58°). Under the P condition, all data points of N2 fell along the diagonal dotted line, indicating a sharply deteriorated localization performance with the data points spread larger on the NH side (gain = 0.12, MAE = 65°).

**FIGURE 5:**
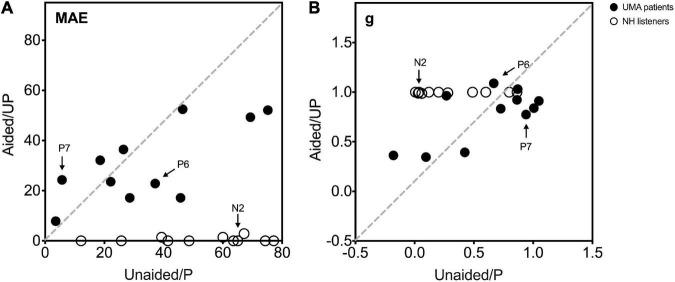
**(A)** The MAE and **(B)** g under monaural listening conditions (unaided and P, Y-axis) are plotted against those under binaural listening conditions (aided and UP, x-axis). Black circle data points indicate 11 UMA children, and white circle data points indicate the controls. The two UMA children and one control depicted in Figure 4 are marked in this figure (P6, P7, and N2). An MAE near 0 and a gain near 1 demonstrate a close-to-normal sound localization performance. Data of participants with the same sound localization performance when listening monaurally and binaurally are displayed on the gray dotted diagonal. A data point below the diagonal in **(A)** and above the diagonal in **(B)** represents a better sound localization performance when listening under binaural conditions than under monaural conditions. UMA, unilateral microtia and atresia; NH, normal hearing group; MAE, mean absolute error; g, response gain; Unaided, the unaided condition of UMA; Aided, the aided condition of UMA; P, the plugged condition of controls; UP, the unplugged condition of controls.

Individual data on sex, age, MAE, gain, bias, and *r*^2^ for all participants are presented in Supplementary Table 1. The MAE and gain under monaural listening conditions (unaided and P) are plotted against those under binaural listening conditions (aided and UP) in Figure 5. Varying sound localization performance was observed in the 11 children with UMA under the unaided condition. When the mean gain and MAE of all children with UMA were compared between the unaided and aided conditions, no significant differences were identified (gain: *p* = 0.104, MAE: *p* = 0.436). Control listeners showed good sound localization performance in the UP condition. All exhibited considerable deterioration after being plugged (gain: *p* < 0.01; MAE: *p* < 0.01), with most of them being unable to localize the stimuli presented from the plugged side. Although no significant difference was observed between the unaided and P conditions (gain: *p* = 0.073; MAE: *p* = 0.073), the results indicated that children with UMA showed better sound localization performance (smaller MAE) than the stimulated acquired UCHL listeners. This phenomenon might be related to the adaptation to congenital unilateral asymmetric hearing loss; however, the benefit of this adaptation was insufficient for children with UMA to localize sound as accurately as the normal controls did.

### Influence of a bone conduction device on sound localization accuracy

To further explore the influence of BCDs on the localization ability of patients with UMA. The localization accuracy of the patients with UMA and the controls were calculated on the impaired (including the atretic and plugged) and the contralateral (including the healthy and unplugged) side, respectively.

**FIGURE 6:**
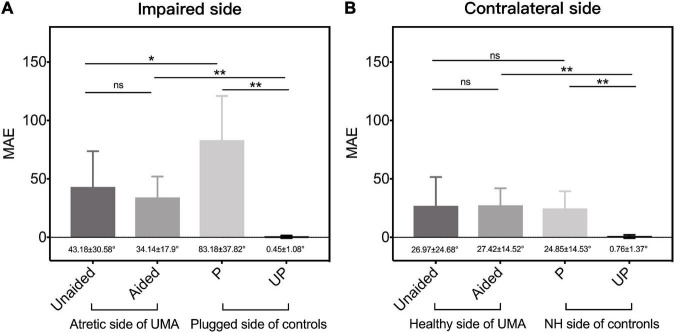
The mean MAE of patients with UMA and the controls, respectively, on **(A)** the impaired (including the atretic side of UMA and the plugged side of controls) side and the **(B)** contralateral (the healthy side of UMA and the unplugged side of controls) side. Error bars represent mean ± *SD*. Significant differences, *p < 0.05, **p < 0.01. ns, not significant; UMA, unilateral microtia and atresia; MAE, mean absolute error; Unaided, the unaided condition of UMA; Aided, the aided condition of UMA; P, the plugged condition of controls; UP, the unplugged condition of controls; NH, normal hearing; *SD*, standard deviation.

A better sound localization accuracy was observed in children with UMA (43.18 ± 30.58° vs. 83.18 ± 37.82°, *p* < 0.05) on the impaired side than in controls in the P condition (Figure 6A). The relative better sound localization accuracy observed in unaided children with UMA, as compared with plugged control listeners, may be attributed to the utilization of distorted remaining binaural cues. For patients aided with a BCD, there was no difference in the MAE between the unaided and aided conditions on the impaired side (43.18 ± 30.58° vs. 34.14 ± 17.9°, *p* = 0.303) or the contralateral side (26.97 ± 24.68° vs. 27.42 ± 14.52°, *p* = 0.79), indicating that the BCD use was not detrimental to the original sound localization ability of the patients with UMA (Figure 6).

### Prediction of the benefits of sound localization accuracy among bone conduction device users

**FIGURE 7:**
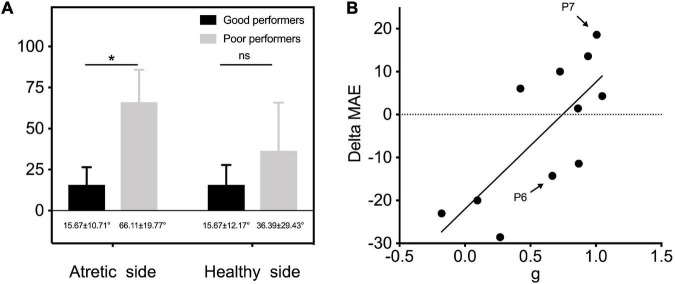
**(A)** MAE outcomes of two subgroups of good performers (*n* = 5; gain > 0.75) and poor performers (*n* = 6; gain ≤ 0.75) were separately compared on the atretic and healthy sides. **(B)** Individual data of gain of UMA children in unaided conditions are plotted as a function of delta MAE between unaided and aided conditions. The linear regression was conducted to explore the predictive effect of gain on the benefits of sound localization accuracy by fitting BCDs. P6 and P7 depicted in Figure 4 are marked in this figure. Delta MAE, aided MAE—unaided MAE. Significant differences, *p < 0.05. ns, not significant; MAE, mean absolute error; g, response gain.

Our results showed that some patients with congenital UCHL have relatively good monaural directional hearing without any hearing intervention (e.g., P7). All 11 children with UMA were divided into two subgroups of good performers (*n* = 5; gain > 0.75) and poor performers (*n* = 6; gain ≤ 0.75) according to the criterion in Agterberg et al.’s (2019) research. When the MAE outcomes were separately compared bilaterally, a significantly better sound localization accuracy was observed in good performers on the atretic side (15.67° ± 10.71° vs. 66.11° ± 19.77°, *p* < 0.05, Figure 7A).

Correlational analysis was further conducted to explore the predictive effect of gain on the benefits of sound localization accuracy by fitting BCDs (delta MAE = aided MAE—unaided MAE, with a smaller delta MAE representing a better improvement in sound localization accuracy). The results revealed an evident relationship between gain and delta MAE (*r*^2^=0.553, *p* < 0.05), indicating that children with UMA who have poor sound localization performance (lower gain) showed more improvement in sound localization after being fitted with BCDs (Figure 7B).

Besides, the Mann-Whitney *U*-test and Spearman correlation analysis regressions were conducted to investigate the influence of sexes, sides of impairment, etiologies, age, and follow-up time on unaided, aided, and delta MAEs in patients with UMA. The results indicated the absence of the main effect (Supplementary Tables 2, 3).

## Discussion

### Hearing benefits of a bone conduction device

In the present study, the ADHEAR system remarkably improved the hearing thresholds and speech perception under quiet and noisy conditions in patients with UMA. Patients with UMA had a mean FHG of 23.73 ± 3.47 dB HL over a frequency range of 0.5–4 kHz; this result lies in the middle of the range of the previously published data of children wearing the ADHEAR system (17–35.6 dB HL) (Dahm et al., 2019; Neumann et al., 2019). Better speech perception abilities were also achieved in quiet and noisy conditions with high participant satisfaction post-BCD-use.

### Sound localization performance of children with unilateral microtia and atresia

Consistent with the findings of previous studies (Agterberg et al., 2019), our results showed an inter-subject variability of directional hearing in children with UMA in unaided conditions. Amongst children with congenital UCHL, good performers might have learned to use remaining binaural difference cues to localize sound sources without hearing amplification, especially when stimuli are presented at an intensity higher than the audibility of the affected ear (Thompson et al., 2020). Another hypothesis for good directional hearing in patients with UCHL is that some of them may rely on monaural cues to achieve good sound localization performance. Vogt et al. (2020) confirmed that patients with congenital UCHL rely on monaural spectral cues to detect high-frequency sound sources by comparing localization accuracy with and without covering the normal hearing ear pinna. Van Wanrooij and Van Opstal (2004) evaluated nine listeners with chronic unilateral hearing loss through a group of broadband sound stimuli fixed at 60 dB, and the results indicated a strong reliance on the ambiguous HSE in familiar acoustic environments. However, no relationship was found between patients’ characteristics and their unaided sound localization performance.

### Influence of a bone conduction device on sound localization accuracy

In our study, no significant improvement in sound localization accuracy was observed in children with UMA aided with BCDs. Similar results have also been obtained in previous studies (Kunst et al., 2008; Weiss et al., 2017) regarding the application of bone-anchored hearing aid and Bonebridge (MED-EL, Innsbruck, Austria) in congenital UCHL. The inability to perform binaural hearing may be a consequence of two factors: first, the hearing asymmetry still exists, as the BCD was not able to produce sufficient intensity input to provide the same hearing threshold as that of the ear with NH; second, the processing time delay and inconsistent stimulation are inherent characteristics of BCD signals, and the BC signals with less reliable and constant cues may also prevent children with congenital UCHL from having restored binaural hearing (Sabin et al., 2005). However, other studies reported improvement in sound localization accuracy when a BCD was used in patients with congenital UCHL (Nelissen et al., 2016; Vyskocil et al., 2017; Vogt et al., 2018). In a recent study involving nine children and adolescents with congenital UMA, better spatial hearing accuracy was found when listening through the Bonebridge, suggesting that this benefit is not based on the processing of binaural cues because the improvement was only observed on the impaired ear side (Vogt et al., 2018). Potential explanations for these conflicting observations may be the age gap of the enrolled patients and methodological differences in the procedure. In summary, it is favorable that sound localization abilities of the intact ear did not deteriorate with the cross-hearing of the BCD use, and this result might be a consequence of the insufficient high-frequency sound transmission of BCDs (Dobrev et al., 2019) and does not interfere with the spectral cues from the contralateral healthy ear.

### Predictive factors for the benefits of sound localization accuracy among bone conduction device users

As shown in Figure 7B, patients with poor unaided spatial hearing (e.g., P6) exhibited more evident improvement (smaller delta MAE) in sound localization accuracy when aided with a BCD. Hence, the original horizontal sound localization performance of listeners with UCHL was a good predictor of their sound localization accuracy under BCD-aided conditions; thus, there is a greater need for early hearing intervention in poor performers who cannot make good use of remaining binaural differences to localize sound sources. As asymmetry hearing induces auditory system reorganization, and animal models of UCHL have shown the structural and functional weakness of the auditory system, thereby affecting binaural hearing integration (Tillein et al., 2016), there seems to be a consensus that early rehabilitation of binaural hearing seems to be better than later rehabilitation (Shirane et al., 2020).

One main limitation of the study is that the factors influencing sound localization accuracy amongst listeners with congenital UCHL are not entirely clear. The small age span in the present study (we mainly included children aged 5–11 years) may be attributed to the absence of a significant correlation between patient characteristics and individual sound localization differentiation. Thus, more factors influencing sound localization performance conflict and the optimal age of BCD use need to be investigated in further studies that include more participants of different ages.

In conclusion, some children with UMA were able to compensate using the remaining distorted binaural cues to detect sound sources, unlike the children with stimulated acquired UCHL; however, this compensating ability was still far worse than children with NH and varied across individuals. As the application of BCD provided a definite benefit on speech recognition abilities and high participant satisfaction, it is recommended that children, particularly those with poor sound localization performance, should be fitted with non-surgical BCDs at an early age.

## Data availability statement

The original contributions presented in this study are included in the article/Supplementary material, further inquiries can be directed to the corresponding author/s.

## Ethics statement

The studies involving human participants were reviewed and approved by the Medical Committee of Beijing Tongren Hospital, Capital Medical University (TRECKY2018-067). Written informed consent to participate in this study was provided by the participants’ legal guardian/next of kin.

## Author contributions

YJL, SZ, CZ, and SG: conceptualization. YJL, PC, and LY: data curation. JY: formal analysis. SZ and CZ: funding acquisition. LY, RR, and YL: methodology. SZ and DW: project administration. YJL, RR, and YL: visualization and writing-original draft. LY, DW, CZ, and SZ: writing-review and editing. All authors contributed to the article and approved the submitted version.

## Abbreviations

NH: normal hearing
UMA: unilateral microtia and atresia
UCHL: unilateral conductive hearing loss
BCD: bone conduction devices
HSE: head shadow effect
BC: bone Conduction
SD: standard deviation
HL: hearing level
SPL: sound pressure level
UP: the unplugged condition
P: the plugged condition
FHG: functional hearing gain
WRS: word recognition score
MSTM: Mandarin Speech Test Materials
SRT: speech reception threshold
SSN: spectrum-shaped noise
SNR: speechto- noise ratio
IOI-HA: the International Outcome Inventory for Hearing Aids
SSQ-P: the Speech, Spatial, and Qualities of Hearing Test for Parent
MAE: mean absolute error
g/gain: response gain
b: response bias
r2: R square
