# Supplementary material for: Characteristics of sound localization in children with unilateral microtia and atresia and predictors of localization improvement when using a bone conduction device
Source: Front Neurosci. 2022 Aug 25;16:973735. doi: 10.3389/fnins.2022.973735 (PMC9461951; doi:10.3389/fnins.2022.973735)
Supplement: Supplementary file 1 [file Table_1.DOCX]

| Supplementary table 1. The MAE, g, b, and r^2^ for all participants | | | | | | | | | | |
| --- | --- | --- | --- | --- | --- | --- | --- | --- | --- | --- |
| Participant number | | Unaided/P | | | |  | Aided/UP | | | |
|  | MAE (°) | | g | b | r^2^ |  | MAE (°) | g | b | r^2^ |
| P1 | 3.57 | | 1.05 | -2.14 | 0.98 |  | 7.86 | 0.91 | -5 | 0.93 |
| P2 | 18.57 | | 0.94 | 5.71 | 0.82 |  | 32.14 | 0.77 | 23.57 | 0.54 |
| P3 | 22.14 | | 0.86 | 3.57 | 0.77 |  | 23.57 | 0.92 | 0.71 | 0.73 |
| P4 | 75.17 | | -0.18 | -2.88 | 0.04 |  | 52.14 | 0.36 | 0.71 | 0.12 |
| P5 | 26.43 | | 0.73 | -23.57 | 0.63 |  | 36.43 | 0.83 | 19.29 | 0.51 |
| P6 | 37.14 | | 0.67 | -31.43 | 0.38 |  | 22.86 | 1.09 | 4.29 | 0.83 |
| P7 | 5.71 | | 1.01 | 2.86 | 0.96 |  | 24.29 | 0.84 | 4.29 | 0.72 |
| P8 | 45.71 | | 0.27 | -30 | 0.1 |  | 17.14 | 0.96 | 12.86 | 0.86 |
| P9 | 69.29 | | 0.1 | -0.71 | 0.01 |  | 49.29 | 0.35 | -2.14 | 0.15 |
| P10 | 46.43 | | 0.42 | 0.71 | 0.17 |  | 52.49 | 0.39 | -12.86 | 0.14 |
| P11 | 28.57 | | 0.87 | 4.29 | 0.67 |  | 17.14 | 1.03 | 0 | 0.87 |
| Mean ± SD | 34.43 ± 23.31 | | 0.61 ± 0.41 | -6.69 ± 14.27 | 0.5 ± 0.27 |  | 30.49 ± 15.35 | 0.77 ± 0.27 | 4.16 ± 10.66 | 0.58 ± 0.31 |
| N1 | 67.14 | | 0.03 | -12.86 | 0 |  | 2.86 | 0.99 | 0 | 0.98 |
| N2 | 65 | | 0.12 | -63.57 | 0.12 |  | 0 | 1 | 0 | 1 |
| N3 | 41.43 | | 0.6 | -38.57 | 0.78 |  | 0 | 1 | 0 | 1 |
| N4 | 12.14 | | 0.8 | -7.86 | 0.91 |  | 0 | 1 | 0 | 1 |
| N5 | 74.3 | | 0.04 | -70 | 0.01 |  | 0 | 1 | 0 | 1 |
| N6 | 48.57 | | 0.49 | -44.29 | 0.64 |  | 0 | 1 | 0 | 1 |
| N7 | 39.29 | | 0.28 | -10.71 | 0.15 |  | 1.43 | 0.99 | 0 | 0.99 |
| N8 | 63.57 | | 0.2 | -52.14 | 0.11 |  | 0 | 1 | 0 | 1 |
| N9 | 60 | | 0.06 | -44.29 | 0.01 |  | 1.43 | 0.99 | 1.429 | 0.99 |
| N10 | 77.14 | | 0.01 | -75.71 | 0 |  | 0 | 1 | 0 | 1 |
| N11 | 25.71 | | 0.86 | -4.29 | 0.59 |  | 0 | 1 | 0 | 1 |
| Mean ± SD | 52.21 ± 20.71 | | 0.32 ± 0.32 | -38.57 ± 26.07 | 0.3 ± 0.35 |  | 0.52 ± 0.96 | 0.998 ± 0.005 | 0.13 ± 0.43 | 1 ± 0.007 |

Unaided, the unaided condition of patients with UMA; Aided, the aided condition of patients with UMA; P, the plugged condition of control listeners; UP, the unplugged condition of control listeners; P1 - P11, patients; N1 - N11, participants with normal hearing; MAE, mean absolute error; g, response gain; b, response bias; r^2^, R square; SD, standard deviation.
