# Supplementary material for: Characteristics of sound localization in children with unilateral microtia and atresia and predictors of localization improvement when using a bone conduction device
Source: Front Neurosci. 2022 Aug 25;16:973735. doi: 10.3389/fnins.2022.973735 (PMC9461951; doi:10.3389/fnins.2022.973735)
Supplement: Supplementary file 2 [file Table_2.DOCX]

| Supplementary table 2. Differences of unaided, aided, and delta MAEs between groups by nominal variables in patients with UMA | | | | | | | | | | | | |
| --- | --- | --- | --- | --- | --- | --- | --- | --- | --- | --- | --- | --- |
|  |  | Unaided MAE | | |  | Aided MAE | | |  | Delta MAE | | |
|  |  | Mean | SD | Sig. (2-tailed) |  | Mean | SD | Sig. (2-tailed) |  | Mean | SD | Sig. (2-tailed) |
| Sex | M | 23.96 | 24.22 | 0.17 |  | 28.47 | 13.75 | 0.59 |  | 1.51 | 15.1 | 0.15 |
|  | F | 47.5 | 16.71 |  |  | 34.02 | 19.53 |  |  | 13.49 | 14.79 |  |
| Side of impairment | R | 36.58 | 23.27 | 0.76 |  | 34.23 | 15.36 | 0.41 |  | 2.35 | 16.21 | 0.74 |
|  | L | 31.86 | 25.81 |  |  | 26 | 15.76 |  |  | 5.86 | 17.67 |  |
| Aetiology | Stenosis | 26.19 | 37.34 | 0.5 |  | 27.15 | 20.86 | 0.682 |  | 0.96 | 19.5 | 0.57 |
|  | Atresia | 37.52 | 18.37 |  |  | 31.74 | 14.35 |  |  | -5.78 | 15.75 |  |

MAE, mean absolute error; SD, standard deviation; UMA, unilateral microtia and atresia; Unaided MAE, the MAE of patients with UMA in the unaided condition; Aided MAE, the MAE of patients with UMA in the aided condition; Delta MAE = Aided MAE - Unaided MAE; M, male; F, female; R, right; L, left. Sig.: the value < 0.05 was set as level of significance.
