# Supplementary material for: Characteristics of sound localization in children with unilateral microtia and atresia and predictors of localization improvement when using a bone conduction device
Source: Front Neurosci. 2022 Aug 25;16:973735. doi: 10.3389/fnins.2022.973735 (PMC9461951; doi:10.3389/fnins.2022.973735)
Supplement: Supplementary file 3 [file Table_3.DOCX]

| Supplementary table 3. Correlations between continuous variables and unaided, aided, and delta MAEs in patients with UMA | | | | | | | | |
| --- | --- | --- | --- | --- | --- | --- | --- | --- |
|  | Unaided MAE | |  | Aided MAE | |  | Delta MAE | |
|  | r^2^ | Sig. (2-tailed) |  | r^2^ | Sig. (2-tailed) |  | r^2^ | Sig. (2-tailed) |
| Age  (years) | 0.18 | 0.19 |  | 0.2 | 0.17 |  | 0.04 | 0.58 |
| Follow-up time (weeks) | 0 | 0.98 |  | 0.01 | 0.75 |  | 0.01 | 0.74 |

MAE, mean absolute error; SD, standard deviation; UMA, unilateral microtia and atresia; Unaided MAE, the MAE of patients with UMA in the unaided condition; Aided MAE, the MAE of patients with UMA in the aided condition; Delta MAE: Aided MAE - Unaided MAE; Sig.: the value < 0.05 was set as level of significance.
